# A novel telomere biology disease‐associated gastritis identified through a whole exome sequencing‐driven approach

**DOI:** 10.1002/cjp2.349

**Published:** 2023-11-22

**Authors:** Namrata Setia, Daniela del Gaudio, Priscilla Kandikatla, Kelly Arndt, Melissa Tjota, Peng Wang, Jeremy Segal, Mir Alikhan, John Hart

**Affiliations:** ^1^ Department of Pathology University of Chicago Chicago IL USA; ^2^ Department of Human Genetics University of Chicago Chicago IL USA; ^3^ NorthShore University Health System Evanston IL USA

**Keywords:** gastritis, *POT1*, *DCLRE1B*, atrophy, whole exome sequencing, telomeropathy

## Abstract

A whole exome sequencing (WES)‐driven approach to uncover the etiology of unexplained inflammatory gastritides has been underutilized by surgical pathologists. Here, we discovered the pathobiology of an unusual chronic atrophic gastritis in two unrelated patients using this approach. The gastric biopsies were notable for an unusual pattern of gastritis with persistent dense inflammation, loss of both parietal and neuroendocrine cells in the oxyntic mucosa, and sparing of the antral mucosa. The patients were found to harbor pathogenic variants in telomeropathic genes (*POT1* and *DCLRE1B*). Clonality testing for one of the patients showed evidence of evolving clonality of TCR‐gene rearrangement. Both patients showed significantly decreased numbers of stem/progenitor cells by immunohistochemistry, which appears to be responsible for the development of mucosal atrophy. No such cases of unusual chronic atrophic gastritis in the setting of telomeropathy have been previously reported. The loss of stem/progenitor cells suggests that stem/progenitor cell exhaustion in the setting of telomere dysfunction is the likely mechanism for development of this unusual chronic atrophic gastritis. The results underscore the need for close monitoring of these gastric lesions, with special regard to their neoplastic potential. This combined WES‐driven approach has promise to identify the cause and mechanism of other uncharacterized gastrointestinal inflammatory disorders.

## Introduction

Outside of the purview of monogenic inflammatory bowel disease‐like pathology, whole exome sequencing (WES) has not been used as an ancillary technique for uncovering etiologies of unexplained severe inflammatory gastric injuries. The pathologic findings of WES can be validated with other methods available to surgical pathologists. Using this approach, we identified the etiology and disease mechanism of a previously undescribed persistent unusual chronic atrophic gastritis in two unrelated patients.

## Materials and methods

### Ethical approval statement

The study protocol was approved by the Institutional Review Board of the University of Chicago (IRB21‐1341) and complied with all provisions of the Declaration of Helsinki, 1975, as revised in 1983.

### WES, Sanger sequencing, next generation sequencing‐based clonality assessment, and immunohistochemical analyses

WES was performed on the biopsy specimens using the NextSeq Illumina platform. Variants in a custom panel of 2,502 genes, which included genes expressed in gastrointestinal tract, B‐ and T‐cells identified from the Human Protein Atlas, and the variants in human gene mutation database were analyzed [1, 2]. Exons and canonical splice sites were identified and evaluated using a validated, custom bioinformatic pipeline. Pathogenic variants were confirmed by Sanger sequencing performed on another sample from the same patient. Next generation sequencing‐based clonality testing was performed using commercially available LymphoTrack assays (Invivoscribe, Inc., SanDiego, CA, USA) [3]. All available pathologic slides were reviewed. Additional immunohistochemical stains for H+/K+ ATPase, Pepsinogen I, TFF2, and Mist1 were performed to assess atrophy and metaplastic changes in the gastric mucosa. Dual immunohistochemical staining for CCK2R/Ki67 and TFF2/Ki67 was performed to highlight the stem/progenitor cell population.

Additional details of the clinical presentation, sequencing, immunohistochemical methods, statistical analysis, and clonality are described in supplementary material, Sections 1–6.

## Results

### Clinical and laboratory findings

**Figure 1:**
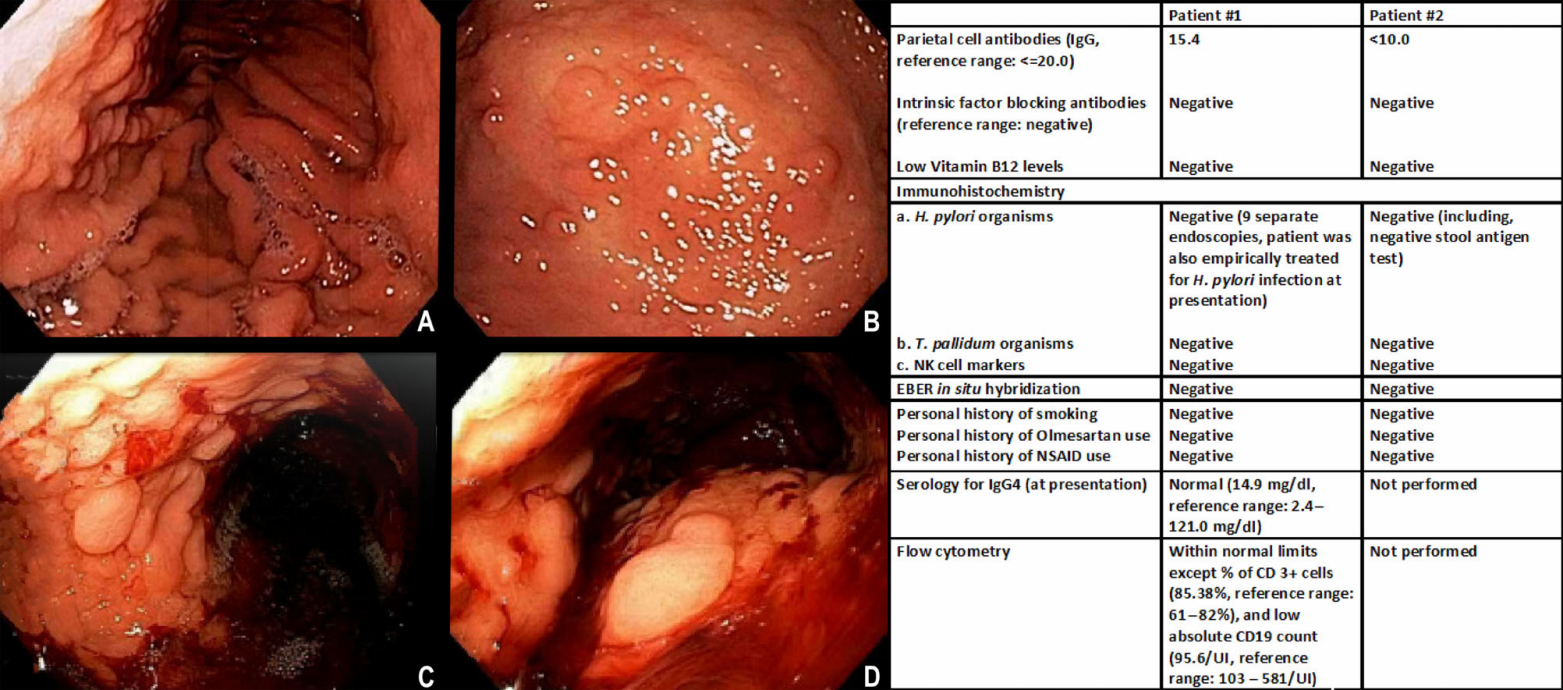
Left panels: (A and B) patient #1 and (C and D) patient #2: representative endoscopic images demonstrate diffuse severe nodularity, friability, and rare erosive lesions in gastric cardiac, fundic, and body mucosa. Right panels: tabulated extensive clinical and laboratory work‐up performed to exclude common and uncommon diagnostic etiologies of gastritis for patient #1 and patient #2.

Patients #1 and #2 were 33‐ and 60‐year‐old females at initial presentation and underwent esophagogastroduodenoscopy for abdominal pain associated with fatigue for 3 years for patient #1 and abdominal pain and diarrhea for patient #2. Patient #1 reported dyspepsia associated with epigastric pain, exacerbated by meals, alcohol, and stress. She had been treated with pantoprazole 40 mg daily and sucralfate for a prolonged but unclear duration. She continued to be surveilled multiple (10) times for epigastric pain over the next 11 years of available clinical follow‐up. Patient #2 reported postprandial abdominal pain associated with bloating, which was precipitated by antibiotics prescribed for sinus infection. Although the clinical plan was to undertake surveillance every 2–3 years, she continued to be surveilled multiple (five) times for ongoing abdominal pain over the next 2 years of available clinical follow‐up. The endoscopic findings for both patients were notable for nodular, granular, and erosive lesions in gastric cardiac, fundic, and body mucosa (Figure 1) with sparing of antral mucosa. Pertinent clinical and laboratory workup is also summarized in Figure 1. Duodenal, ileal, and colorectal biopsies did not show diagnostic abnormality for both patients. Both patients underwent genetic counselling and testing with commercial multigene panels, which were negative for clinically significant variants. The indication for genetic testing for patient #1 was a family history of ovarian cancer, and for patient #2 was a personal history of melanomas (left shin and neck), multiple basal cell carcinomas involving her left eyelid, left arm, and chest (*n* = 46), pituitary microadenoma, and a family history of breast cancer, lymphoma, lung cancer, melanoma, and pancreatic cancer.

### Histologic presentation

**Figure 2:**
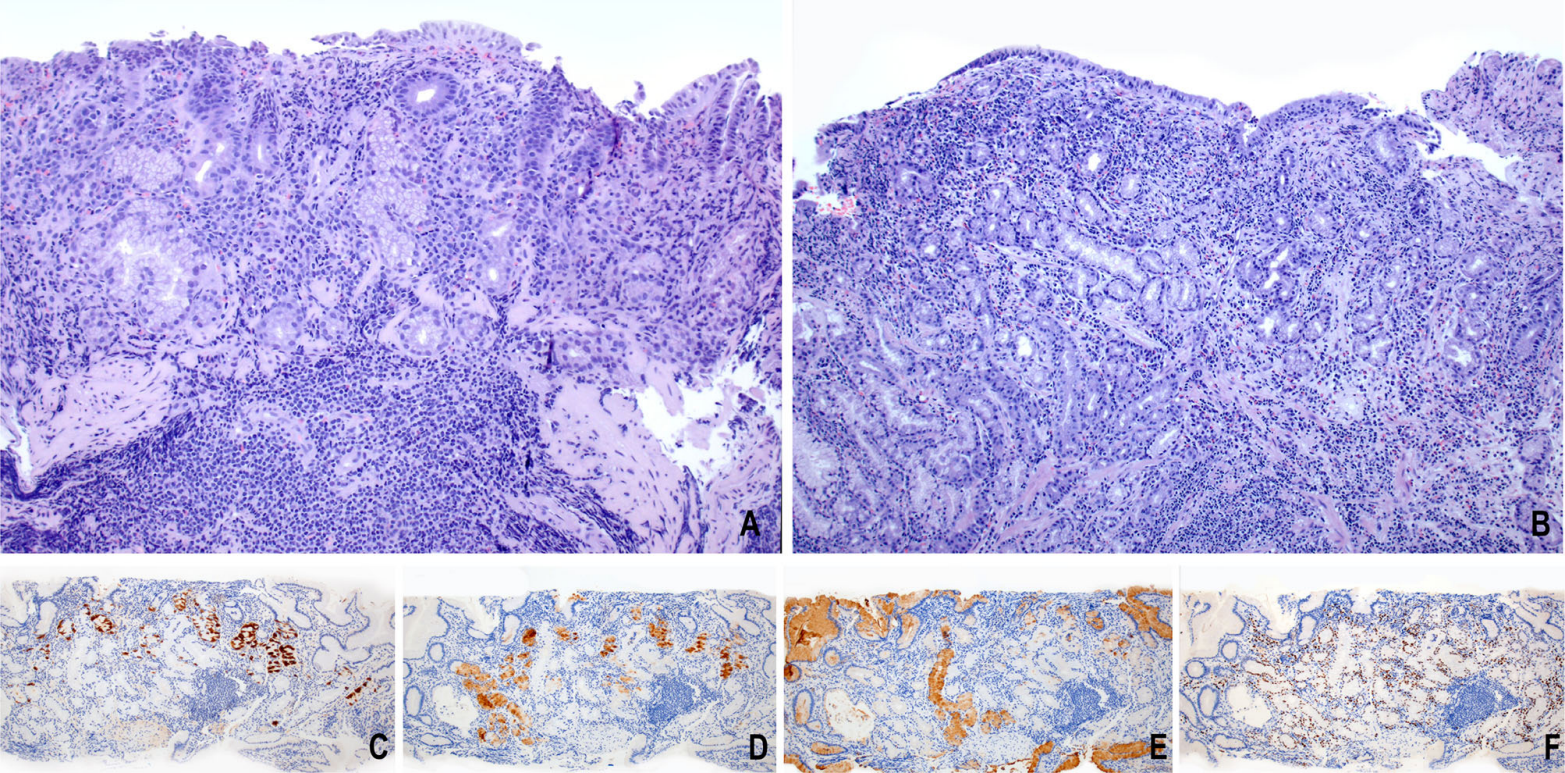
(A) Patient #1 and (B) patient #2: representative H&E images of chronic atrophic gastritis in the oxyntic mucosa with loss of parietal cells, absence of neuroendocrine cell hyperplasia, and prominent dense inflammation. No intestinal metaplasia is present. (C–F) Representative immunohistochemical stains for patient #1: (C) H+/K+ ATPase showing significant loss of parietal cells; (D) Pepsinogen I highlights metaplastic glands with Pepsinogen I expression as indicated by cytoplasmic brown staining; these findings are consistent with pseudopyloric metaplasia; TFF2 (E) and Mist1 (F) show a metaplastic pyloric gland with TFF2 expression as indicated by cytoplasmic brown staining (E) and concurrent loss of nuclear Mist1 expression (F); these findings are consistent with focal spasmolytic polypeptide‐expressing metaplasia.

Gastric mapping biopsies performed from both patients showed severe loss of parietal cells (H+/K+ ATPase−), pyloric (Pepsinogen I−) and pseudopyloric metaplasia (Pepsinogen I+), and absence of enterochromaffin‐like (ECL) cell hyperplasia in the gastric cardiac/fundic and body mucosa. A prominent inflammatory infiltrate was present, which involved the entire mucosa with focal extension through the muscularis mucosa in patient #1. Accompanying intestinal metaplasia was absent. The antral mucosa showed minimal to no involvement by inflammation. Focal prominence of subepithelial collagen was present. The histologic findings, although within the spectrum of autoimmune gastritis (AIG) due to parietal cell loss and antral sparing, were unusual and inconsistent with conventional diagnostic criteria for AIG due to lack of ECL cell hyperplasia despite severe loss of parietal cells [4]. The metaplastic cells were focally TFF2+ and Mist1−, consistent with spasmolytic polypeptide‐expressing metaplasia. Representative histologic findings are shown in Figure 2.

### Defects in telomere/shelterin‐complex genes identified by WES

**Figure 3:**
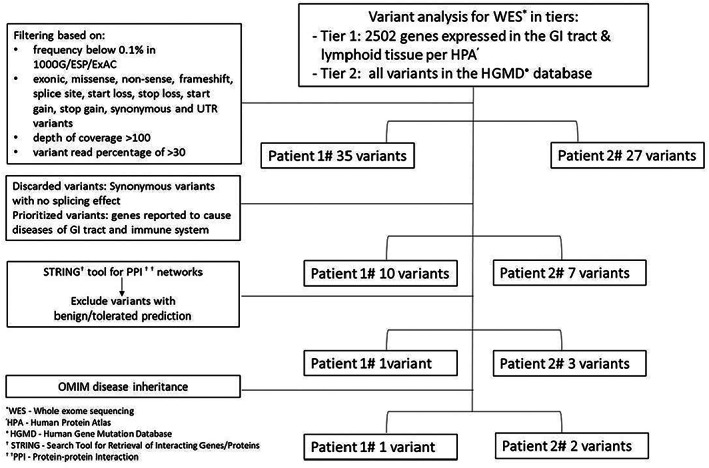
Tiered filtering strategy for the variants identified by WES.

WES of the FFPE biopsy specimens provided 327.59 and 174.22 average depth of coverage, and 98.70% and 98.60% of exome covered for patient #1 and patient #2, respectively. The WES data were analyzed in tiers (Figure 3). The output, after filtering, included 35 variants for patient #1 and 27 variants from patient #2. Variants in genes reported to cause diseases of gastrointestinal tract and immune system were prioritized for analysis. The lists of genes generated for both patients were compared to identify those in the same functional interactive pathways using the STRING tool (v11.5) and assessed for reported disease inheritance in OMIM database [5, 6]. Active interaction sources were limited to ‘*Homo sapiens*’ and an interaction score >0.4 was applied to construct the protein–protein interaction networks (supplementary material, Section 4). After the filtration steps, three variants from two genes in telomere/shelterin‐complex were identified; these included *POT1* (autosomal dominant inheritance) c.1164‐1G>A (splice site, VAF 48.86%) in patient #1, and *DCLRE1B* (alias symbol *APOLLO*, autosomal recessive inheritance) c.1013_1031del (exon), p.Trp338* (VAF 42.63%) and c.781dup (exon), p.Ile261Asnfs*7 (VAF 51.59%) in patient #2. These pathogenic variants were confirmed by Sanger sequencing performed on an additional sample from each patient, which is consistent with germline inheritance (supplementary material, Section 3 and Figures S1 and S2).

### Stem/progenitor cell depletion as proposed etiology and evidence of evolving clonality

**Figure 4:**
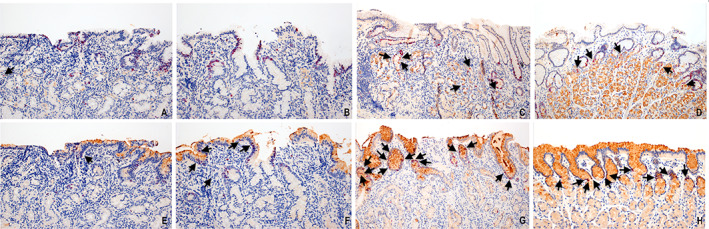
Comparison of dual staining cells marked by CCR2R+ and Ki67+ (top panel) and TFF2+ and Ki67+ (bottom panel), majority marked by black arrows, for patient #1 (A and E), patient #2 (B and F), AIG (C and G), and normal oxyntic mucosa (D and H).

We then sought to determine whether stem/progenitor cell lineage was affected by a telomere/shelterin‐complex gene defect. For this, the number of dual CCK2R+/Ki67+ and dual TTF2+/Ki67+ cells in the isthmus was counted and averaged/mm^2^ for all affected biopsy fragments. These results were compared with randomly selected age‐ and sex‐matched biopsy controls with classic AIG and normal gastric oxyntic mucosa. The average number of dual CCK2R+/Ki67+ cells/mm^2^ was 3 ± 1 in patient #1 and 2 ± 1 in patient #2, compared to 12 ± 6 in AIG and 16 ± 5 in gastric oxyntic mucosa without diagnostic abnormality (wda) (*p* < 0.01, patient #1 and #2 versus AIG and patient #1 and #2 versus wda). The average number of dual TFF2+/Ki67+ cells/mm^2^ was 4 ± 2 in patient #1 and 8 ± 3 in patient #2, compared to 30 ± 6 in AIG and 12 ± 5 in gastric oxyntic mucosa wda (*p* < 0.01, patient #1 and #2 versus AIG, and *p* < 0.05, patient #1 and #2 versus wda) (Figure 4). Immunohistochemical staining for Mist1 did not show increased staining in the isthmic region. Due to significant inflammation, we performed clonality testing which showed evolution of TCR‐gene rearrangement at year 9, which was not present at year 7 for patient #1 (supplementary material, Section 7 and Figures S4–S6). However, there was no evidence of lymphoma by immunohistochemical expression of CD markers (supplementary material, Section 7). The second patient, last seen at year 2, did not show a B/TCR clonal rearrangement (supplementary material, Section 7 and Figure S7).

## Discussion

Here, we identified pathogenic variants in *POT1* and *DCLRE1B* (alias symbol *APOLLO*) in two unusual severe gastritides thought to be of unknown etiology despite extensive clinical workup. Unlike other telomere complex genes which cause shortening of telomere length, these genes are associated with long and normal telomere length, respectively [7, 8, 9]. Data regarding involvement of *POT1* and *DCLRE1B* genes in telomeropathies are only recently emerging with proposals that these genes should be included on cancer‐risk predisposing multigene panel tests as well as genetic tests for telomere biology dysfunction [10, 11]. As has been observed, the affected individuals may not present with the typical triad of short telomere syndrome, particularly in adults, and there has been a recent proposal to use ‘telomere biology disorder (TBD)’ instead of ‘short telomere syndrome’ to allow such contradictions to be addressed [7].

Our investigation revealed a relative depletion of isthmic progenitor/stem cells in the gastric biopsies. Sheng *et al* demonstrated that CCK2R+ progenitor cells are short‐lived progenitors that divide and supply daughter cells for a few cycles and replenish ECL and parietal lineages [12]. On the contrary, TFF2+ cells above the neck region are the progenitors for parietal cells but not ECL cell lineages. Based on our investigation, we propose this unusual gastritis is a manifestation of susceptibility of these progenitors/stem cells in individuals with telomere gene defects. Manifestation in the gastric corpus may be explained by the unique location of the stem cell niche in oxyntic mucosa (near the lumen rather than the base) unlike the antrum and the rest of the gastrointestinal tract, allowing more exposure to surface irritants [13].

TBDs are emerging multisystem cancer‐predisposition syndromes associated with a higher risk of benign and malignant hematolymphoid and solid neoplasms, described particularly in the context of *POT1* genetic mutations [14, 15, 16, 17, 18, 19]. More recently, genetic anticipation has been described in successive generations with heterozygous *POT1* gene defects [14]. Given the rarity and variable clinical presentation of TBD‐associated cancer‐predisposition syndromes, the mechanism behind the predisposition is not well defined. Histologic evidence of lymphoproliferation and evolving clonality in patient #1 and the concerning personal and family history of multiple neoplasms in patient #2 highlight the importance of identifying these genetic defects. These efforts will eventually allow for the development of screening and treatment guidelines for patients with TBDs.

## Author contributions statement

NS and JH conceived and designed the study. DdG, PK, KA, MT, PW, JS and MA provided technical support. NS and DdG collected and/or assembled data. NS, DdG and MA analyzed and interpreted data. NS wrote the manuscript. DdG, MT, PW, JS, MAK and JH participated in review and final approval of the manuscript.

## Supporting information


**Section 1.** Clinical presentation (additional information)
**Section 2.** Whole exome sequencing
**Section 3.** Sanger sequencing (including Figures S1 and S2)
**Section 4.** String analysis (including Figure S3)
**Section 5.** Antibody information and antibody protocols for immunohistochemistry
**Section 6.** Statistical analysis
**Section 7.** Clonality by next‐generation sequencing and lymphoma work‐up (including Figures S4–S7)
**Figure S1.** Sanger sequencing results for *DCLRE1B* variant c.781het_dupA and *DCLRE1B* variant c.1013_1031del variant
**Figure S2.** Sanger sequencing results for *POT1* variant c.1164‐1G>A
**Figure S3.** STRING database analysis for the resultant 16 genes (17 variants) (cited only in supplementary material)
**Figure S4.** LymphoTrack TRG analysis of V‐J usage and V‐J sequence frequencies performed on the sample obtained at year 9 from patient #1
**Figure S5.** LymphoTrack TRG analysis of V‐J usage and V‐J sequence frequencies performed on the sample obtained at year 7 from patient #1
**Figure S6.** LymphoTrack IGH analysis performed on the sample obtained at year 9 from patient #1
**Figure S7.** LymphoTrack IGH and TRG analysis performed on the last sample from patient #2

## Data Availability

The authors confirm that the data supporting the findings are available within the article and its supplementary materials.
