# Supplementary material for: A novel telomere biology disease‐associated gastritis identified through a whole exome sequencing‐driven approach
Source: J Pathol Clin Res. 2023 Nov 22;10(1):e349. doi: 10.1002/cjp2.349 (PMC10766041; doi:10.1002/cjp2.349)
Supplement: Supplementary file 1 — Section 1. Clinical presentation (additional information) Section 2. Whole exome sequencing Section 3. Sanger sequencing (including Figures S1 and S2) Section 4. String analysis (including Figure S3) Section 5. Antibody information and antibody protocols for immunohistochemistry Section 6. Statistical analysis Section 7. Clonality by next‐generation sequencing and lymphoma work‐up (including Figures S4–S7) Figure S1. Sanger sequencing results for DCLRE1B variant c.781het_dupA and DCLRE1B variant c.1013_1031del variant Figure S2. Sanger sequencing results for POT1 variant c.1164‐1G>A Figure S3. STRING database analysis for the resultant 16 genes (17 variants) (cited only in supplementary material) Figure S4. LymphoTrack TRG analysis of V‐J usage and V‐J sequence frequencies performed on the sample obtained at year 9 from patient #1 Figure S5. LymphoTrack TRG analysis of V‐J usage and V‐J sequence frequencies performed on the sample obtained at year 7 from patient #1 Figure S6. LymphoTrack IGH analysis performed on the sample obtained at year 9 from patient #1 Figure S7. LymphoTrack IGH and TRG analysis performed on the last sample from patient #2 [file CJP2-10-e349-s001.pdf]

A novel telomere biology disease-associated gastritis identified through a whole exome sequencing-driven approach

N Setia *et al*., *J Pathol Clin Res,* https://doi.org/10.1002/cjp2.349

Supplementary material: Sections 1–7

Supplementary Figures S1–S7

Reference numbers refer to the main text list.

Section 1. Clinical Presentation (additional information)

Additional workup performed for patient #1 included gadolinium enhanced MRI/MRCP of the abdomen to exclude a gastrinoma, which was negative. Additional workup performed for patient #2 included normal gastric emptying studies, exclusion of amyloidosis, a negative stool GI Panel PCR test.

Section 2. Whole Exome Sequencing

Genomic DNA was extracted from formalin-fixed, paraffin-embedded (FFPE) tissue using the QIAamp DNA FFPE Tissue Kit (Qiagen, Valencia, CA). DNA quantification was performed using a Qubit fluorometric assay (Thermo Fisher Scientific, Foster City, CA) and a quantitative PCR assay (hgDNA Quantitation and QC kit; Kapa Biosystems, Wilmington, MA). FFPE specimens were prepared using the xGen™ cfDNA & FFPE DNA Library Prep for whole exome sequencing (Integrated DNA Technology, Coralville, IA) according to manufacturer's instructions. Whole exome sequencing library preparation and hybridization capture using the IDT xGen DNA Library prep EZ kit (Integrated DNA Technology, Coralville, IA). Paired end sequencing was performed on the Illumina NovaSeq 6000 platform (Illumina, San Diego, CA, USA) using 150 bp reads to an average depth of coverage of 150x on the genomic DNA.

Sequencing data was converted to FASTQ format and demultiplexed using the bcl2fastq software from Illumina. Paired-end reads were aligned to the GRCh37/hg19 reference genome using the Burrows-Wheeler Alignment BWA-MEM algorithm (arXiv:1303.3997). BAM files were processed, and variants called using GATK's Haplotype Caller best practices pipeline, including

duplicate reading marking and base quality score recalibration [20]. Raw variant calls were filtered based on various quality metrics such as depth, quality by depth score and directional bias. Variants were annotated based on their position in transcript of interest, position relative to the coding sequence, consequence for the protein or mRNA and a collection of direct and indirect evidentiary tools and databases including the Genome Aggregation Database (gnomAD), the Human Gene Mutation Database (HGMD) and a number of in silico tools to predict variant pathogenicity.

Section 3. Sanger Sequencing

The *DLCRE1B* (Ref Seq NM_022836.4) c. 781dup and the c.1013_1031del variants and the *POT1* (Ref Seq NM_015450.3) c.1164-1G>A variant were confirmed via Sanger sequencing with PCR primers flanking the regions of interest (*DLCRE1B* c. 781dup Forward – 5'-TCTACAGCCTGGGAAAGGA- 3', Reverse – 5'- ACTGTCCTGAAAGCCTCCAC-3', *DLCRE1B* c.1013_1031del Forward –5'- TCCACGTCATCCCTTACTCTG-3'; Reverse –5'-GAAGGCTGCTTTTCAAGGTC-3', *POT1* c.1164-1G>A Forward – 5'-TGTTCTGCAAAGTGAATTATCTCC-3', Reverse – 5'-CGTCCTTTTTGATTTTTAGTGGTC-3' all with M13 tails). Sequencing was performed on an ABI3730xl DNA Analyzer (Thermo Fisher Scientific, Inc.) and the results were analyzed using the MutationSurveyor software (SoftGenetics, Inc.). The results are shown in Figures S1 and S2.

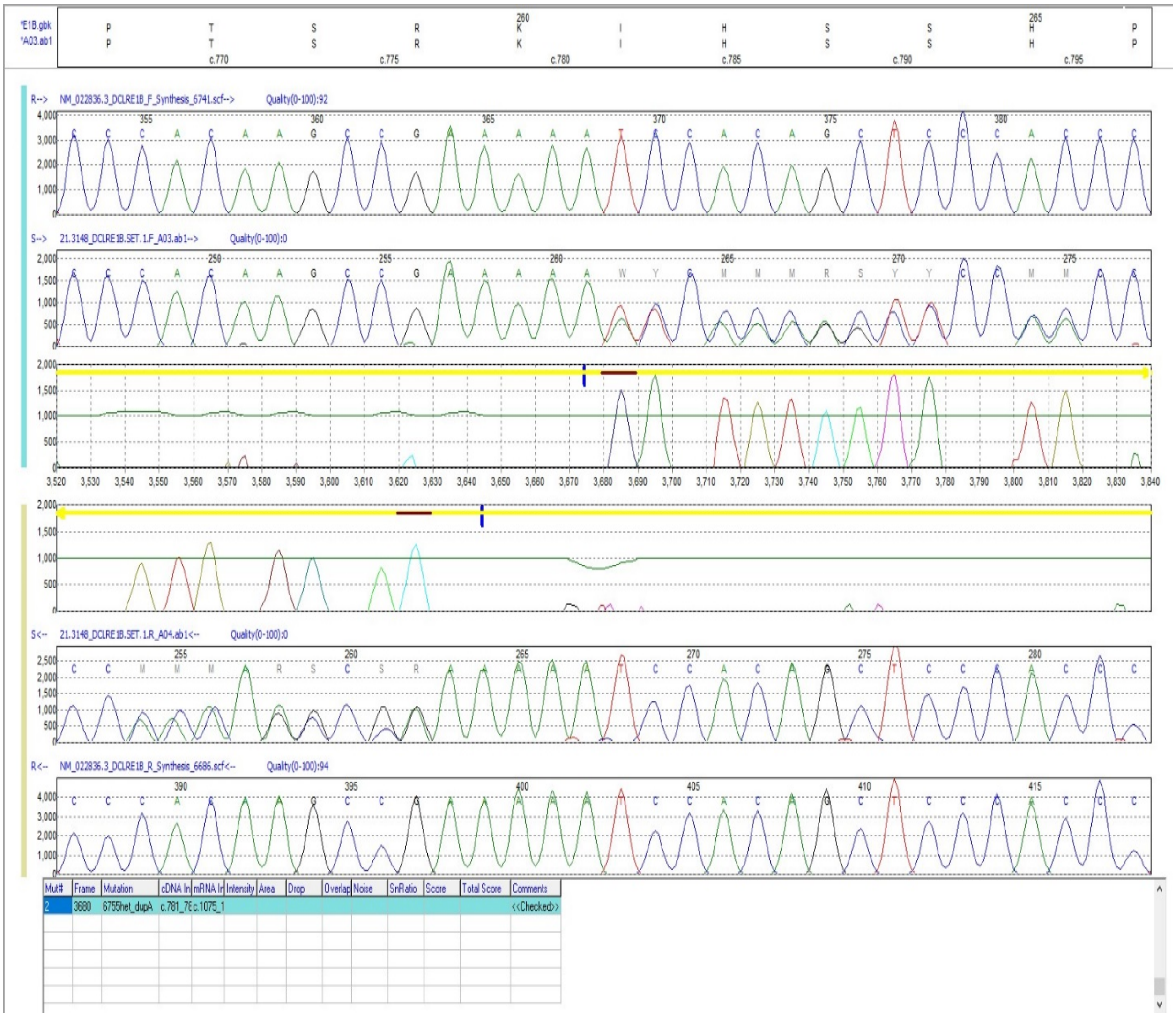

Figure S1A. Sanger sequencing results for *DCLRE1B* variant c.781het_dupA.

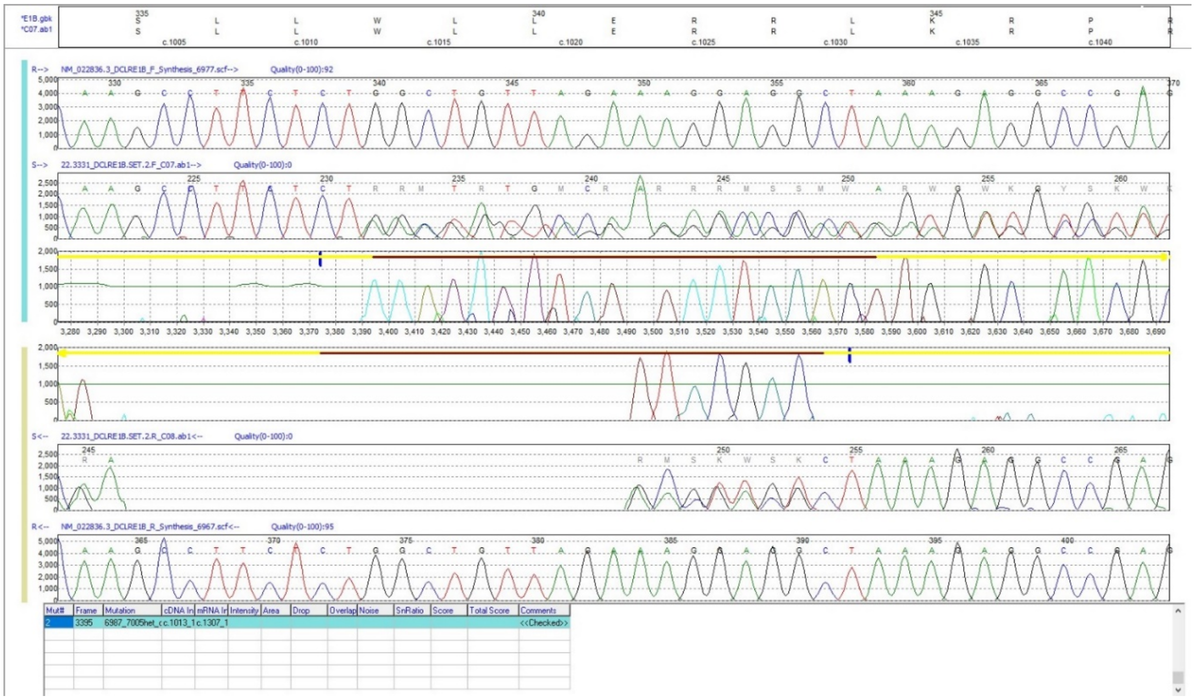

Figure S1B. Sanger sequencing results for *DCLRE1B* variant c.1013_1031del.

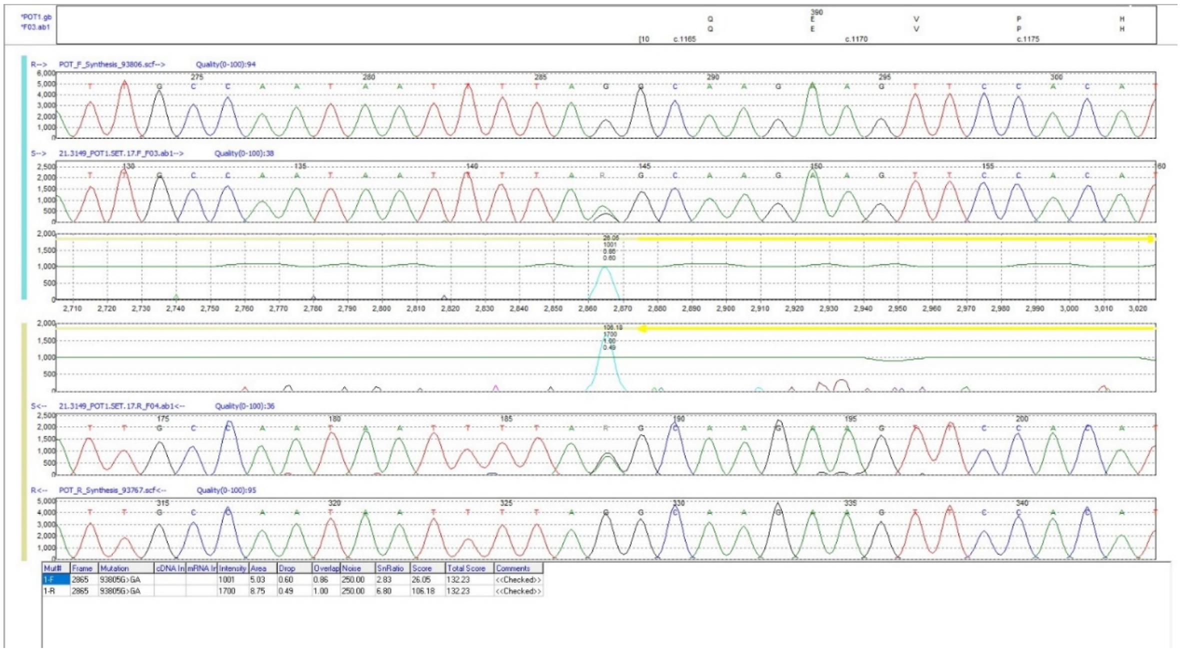

Figure S2. Sanger sequencing results for *POT1* variant c.1164-1G>A.

Of note, the WES did not reveal any pathogenic/likely pathogenic variants in the *MEN1* gene.

Section 4. String Analysis

Search Tool for the Retrieval of Interacting Genes/Proteins (STRING) (https://string-db.org) database was employed to identify common pathologic cluster of proteins (genes) contributing to the pathogenesis of unusual gastritis from the list identified after Tier1 and Tier2 filtering steps, excluding genes with synonymous variants and no splicing effect, and prioritizing variants in genes reported to cause diseases of GI tract and immune system. The resultant 16 genes (17 variants) were analysed using STRING database, which integrates known and predicted protein-protein interactions (PPIs) using information from experimental data, computational prediction methods and public text collections (Figure S3). Active interaction sources, including experiments, databases, text mining, co-occurrence, and co-expression as well as species limited to "*Homo sapiens*" and an interaction score > 0.4 were applied to construct the PPI networks. K-mean clustering identified 3 clusters. The highest interaction score was present between nodes *POT1* and *DCLRE1B* (score 0.985, range 0.406-0.968). Local network cluster (STRING) was enriched for one single cluster (*POT1*, *DCLRE1B* and *CTC1*) with a strength of 2.45 (strength range for functional enrichments: 0.49-2.45) and a false discovery rate of 0.0011.

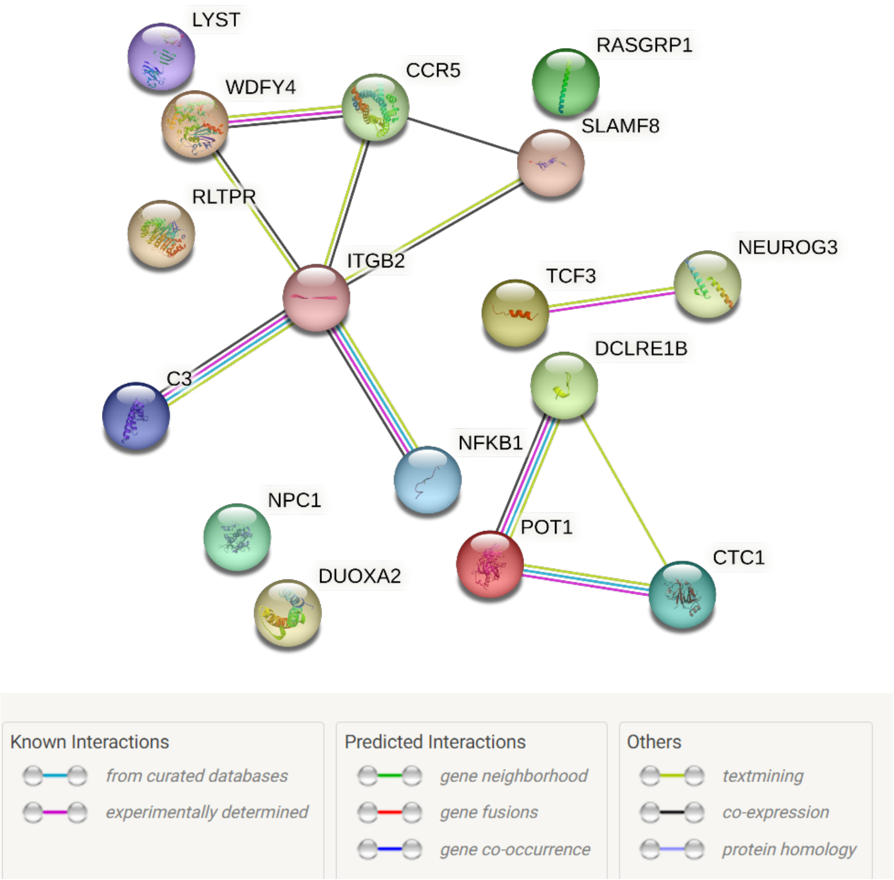

Figure S3. STRING database analysis for the resultant 16 genes (17 variants).

Section 5. Antibody Information and Antibody Protocols for Immunohistochemistry

a. Protocol for anti-H+/K+ ATPase Beta (Santa Cruz, catalog# sc-374094 (C-4), mouse monoclonal, 0.2 mg/ml) on human FFPE GI tissues: The slide was stained using Leica Bond RX automated stainer. After deparaffinization and rehydration, tissue section was heat treated for 20 minutes with antigen retrieval solution (Leica Biosystems, AR9961). Anti- H+/K+ ATPase Beta antibody (1:2000) was applied on tissue sections for one-hour incubation at room temperature and the antigen-antibody binding was detected with Bond Polymer Refine Detection (Leica Biosystems, DS9800). Tissue sections were briefly immersed in hematoxylin for counterstaining and were covered with cover glasses.

b. Protocol for anti-Pepsinogen 1 (Bio-Rad, catalog# 7240-1009, mouse monoclonal, clone: 8003(99/12), 0.5 mg/ml) on human FFPE GI tissues: The slide was stained using Leica Bond RX automated stainer. After deparaffinization and rehydration, tissue section was heat treated

for 20 minutes with antigen retrieval solution (Leica Biosystems, AR9961). Anti-Pepsinogen 1 antibody (1:400) was applied on tissue sections for one-hour incubation at room temperature and the antigen-antibody binding was detected with Bond Polymer Refine Detection (Leica Biosystems, DS9800). Tissue sections were briefly immersed in hematoxylin for counterstaining and were covered with cover glasses.

c. Protocol for anti-TFF2 (ProteinTech Group, catalog# 13681-1-AP, rabbit polyclonal, 400 μg/ml) on human FFPE GI tissues: The slide was stained using Leica Bond RX automated stainer. After deparaffinization and rehydration, tissue section was heat treated for 20 minutes with antigen retrieval solution (Leica Biosystems, AR9961). Anti-TFF2 antibody (1:4000) was applied on tissue sections for one-hour incubation at room temperature and the antigen-antibody binding was detected with Bond Polymer Refine Detection (Leica Biosystems, DS9800) without Post Primary reagent. Tissue sections were briefly immersed in hematoxylin for counterstaining and were covered with cover glasses.

d. Protocol for anti-Mist1 (Cell Signaling Technology, catalog#14896S, rabbit monoclonal, clone D7N4B) on human FFPE GI tissues: The slide was stained using Leica Bond RX automated stainer. After deparaffinization and rehydration, tissue section was heat treated for 20 minutes with antigen retrieval solution (Leica Biosystems, AR9640). Anti-Mist1 antibody (1:100) was applied on tissue sections for one-hour incubation at room temperature and the antigen-antibody binding was detected with Bond Polymer Refine Detection (Leica Biosystems, DS9800) without Post Primary reagent. Tissue sections were briefly immersed in hematoxylin for counterstaining and were covered with cover glasses.

e. Protocol for anti-CCKBR/Cholecystokinin-BR/(Norus Bio, Cat#NB100-2803, goat polyclonal antibody, 0.5 mg/ml) and Ki67 (RM-9106-AS1, ThermoFisher, rabbit monoclonal, clone SP6) on human GI paraffin tissues: The slides were stained on Leica Bond RX Automatic Stainer. After antigen retrieval treatment (epitope retrieval solution II AR9640, Leica Biosystems) for 20 minutes, anti-CCKBR antibody (1:200) was applied on tissue sections for 60 minutes incubation and the antigen-antibody binding was detected with (ImmPress anti-goat polymer HRP, MP-7405) and DAB (Bond Polymer Refine Detection). The tissue sections were counterstained with hematoxylin and covered with cover glasses. (CCKBR: brown cytoplasmic stain, Ki67: red nuclear staining)

f. Protocol for anti-TFF2 (ProteinTech Group, catalog# 13681-1-AP, rabbit polyclonal, 400 μg/ml) and Ki67 (RM-9106-AS1, ThermoFisher, rabbit monoclonal, clone SP6) on human GI

paraffin tissues: The slide was stained using Leica Bond RX automated stainer. After deparaffinization and rehydration, tissue section was heat treated for 20 minutes with antigen retrieval solution (Leica Biosystems, AR9961). Anti-TFF2 antibody (1:4000) was applied on tissue sections for one-hour incubation at room temperature and the antigen-antibody binding was detected with Bond Polymer Refine Detection (Leica Biosystems, DS9800) without Post Primary reagent. Tissue sections were briefly immersed in hematoxylin for counterstaining and were covered with cover glasses. Before the second antigen-antibody reaction, epitope retrieval solution II (Leica Biosystems, AR9640) was used for 10 minutes. Anti-Ki67 (1:400) was applied on tissue section of 60 minutes incubation and the antigen-antibody binding was detected with Bond red polymer refine detection (Leica Biosystems, DS9390). The tissue sections were covered with cover glasses. The tissue sections were counterstained with hematoxylin and covered with cover glasses. (TFF2: brown cytoplasmic stain, Ki67: red nuclear staining)

Given the reported inconspicuousness of Lrg5+ stem cells in oxyntic mucosa, immunohistochemical assessment for Lrg5+ was not performed.

Section 6. Statistical analysis

The mean values obtained from dual immunohistochemistry between the patients, autoimmune gastritis and biopsies without diagnostic abnormality were compared by one-way analysis of variance with Tukey's honest significant difference post hoc test.

Section 7. Clonality by Next-Generation Sequencing and Lymphoma Work-up

Clonal TRG and IGH V-J rearrangement analysis was performed by next-generation sequencing using the LymphoTrack3 NGS assay (Invivoscribe, San Diego, CA) as previously described following the manufacturer's protocol. The technique employs PCR amplification using master mixes for conserved framework region 1, 2 and 3 (FR1, FR2, and FR3) within the VH region and the JH region for IGH and all conserved regions within the V and the J gene regions described in lymphoid malignancies for TRG with primers derived from barcoded sequence adaptors. The library sequencing was performed using the IonTorrent platform. The output is analysed using LymphoTrack® PGM Software (Invivoscribe, Inc.). Determination of clonality in these cases was based on criteria outlined previously [3]. Overall, the dominant sequences should be >2.5% of top merged reads, and >10-times the polyclonal background.

For patient #1 (Figure S4), TRG analysis on the last sample showed two dominant clones Vg4 Jg1/2 at 20.2% (85,501 reads) and Vg9 Jg1/2 at 38.7% (77,942 reads) in an otherwise polyclonal background (total read count 422,557). These clones were seen at a low level Vg4

Jg1/2 at 18.77% (11,937 reads) and Vg9 Jg1/2 at 6.19% (3,934 reads) in an otherwise polyclonal background (total read count 63,579) (Figure S5). The last sample showed a polyclonal IGH (204,929 reads, Figure S6).

Histologic specimen corresponding to the TCR gene rearranged (with two dominant clones) showed nodular infiltrates of small to medium sized lymphocytes. The infiltrate focally appeared to extend rather deep below the mucosa. The nodular infiltrate was composed of mixed chronic and active inflammation, including eosinophils. Areas of glandular destruction increased intraepithelial lymphocytes, angiocentricity or angiodestructive pattern of growth and necrosis were not seen. Immunohistochemical and in situ hybridization stains performed showed: CD3 positive in the large majority of the lymphoid infiltrate with one apparent follicle showing negative CD3 staining in central cells, consistent with B-cells, CD20 positive in the minority of lymphoid infiltrate, positive B-cells cluster in apparent follicles with some scattered positive larger B-cells. Immunohistochemical stain for AE1/AE3 highlighted the epithelial layer/glands with no prominent apparent intraepithelial lymphocytes, CD138 highlighted scattered positive plasma cells, mostly present between glands and only rarely staining seen in the nodular infiltrate and IgG/IgG4: IgG highlighting plasma cells, similar to CD138. Few scattered IgG4+ plasma cells were also noted, insufficient for a diagnosis of IgG4-related disease. H. pylori stain was negative with valid external positive control and Kappa/lambda ISH: K:L ratio was 2-3:1 Overall, the findings were consistent with reactive lymphoid infiltrate in the upper/mid body.

A

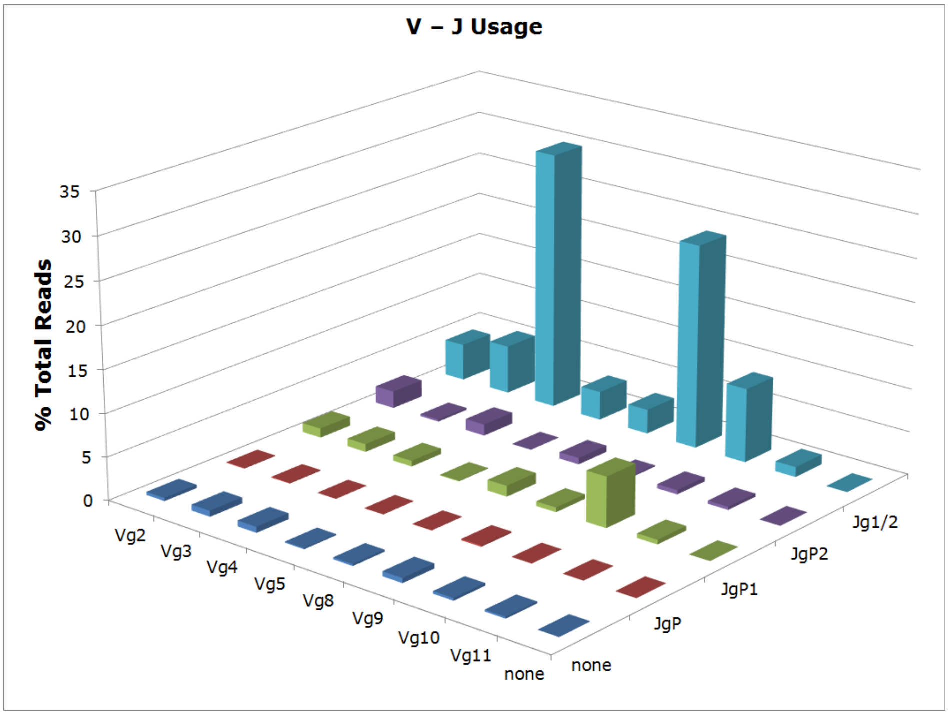
V – J Usage
% Total Reads
35
30
25
20
15
10
5
0
Vg2
Vg3
Vg4
Vg5
Vg8
Vg9
Vg10
Vg11
none
none
JgP
JgP1
JgP2
Jg1/2

B

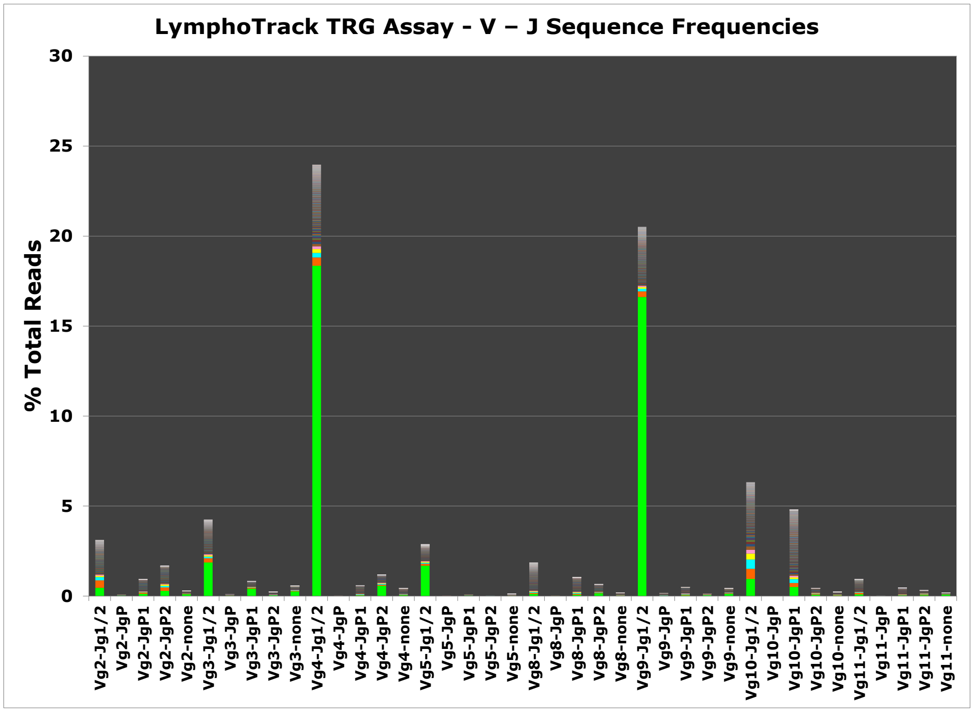
LymphoTrack TRG Assay - V – J Sequence Frequencies
% Total Reads
30
25
20
15
10
5
0
Vg2-Jg1/2
Vg2-JgP
Vg2-JgP1
Vg2-JgP2
Vg2-none
Vg3-Jg1/2
Vg3-JgP
Vg3-JgP1
Vg3-JgP2
Vg3-none
Vg4-Jg1/2
Vg4-JgP
Vg4-JgP1
Vg4-JgP2
Vg4-none
Vg5-Jg1/2
Vg5-JgP
Vg5-JgP1
Vg5-JgP2
Vg5-none
Vg8-Jg1/2
Vg8-JgP
Vg8-JgP1
Vg8-JgP2
Vg8-none
Vg9-Jg1/2
Vg9-JgP
Vg9-JgP1
Vg9-JgP2
Vg9-none
Vg10-Jg1/2
Vg10-JgP
Vg10-JgP1
Vg10-JgP2
Vg10-none
Vg11-Jg1/2
Vg11-JgP
Vg11-JgP1
Vg11-JgP2
Vg11-none

Figure S4. LymphoTrack TRG analysis of (A) V-J usage and (B) V-J sequence frequencies performed on the sample obtained at year 9 from patient #1 showing two dominant clones Vg4 Jg1/2 at 20.2% (85,501 reads) and Vg9 Jg1/2 at 38.7% (77,942 reads) in an otherwise polyclonal background (total read count 422,557).

A

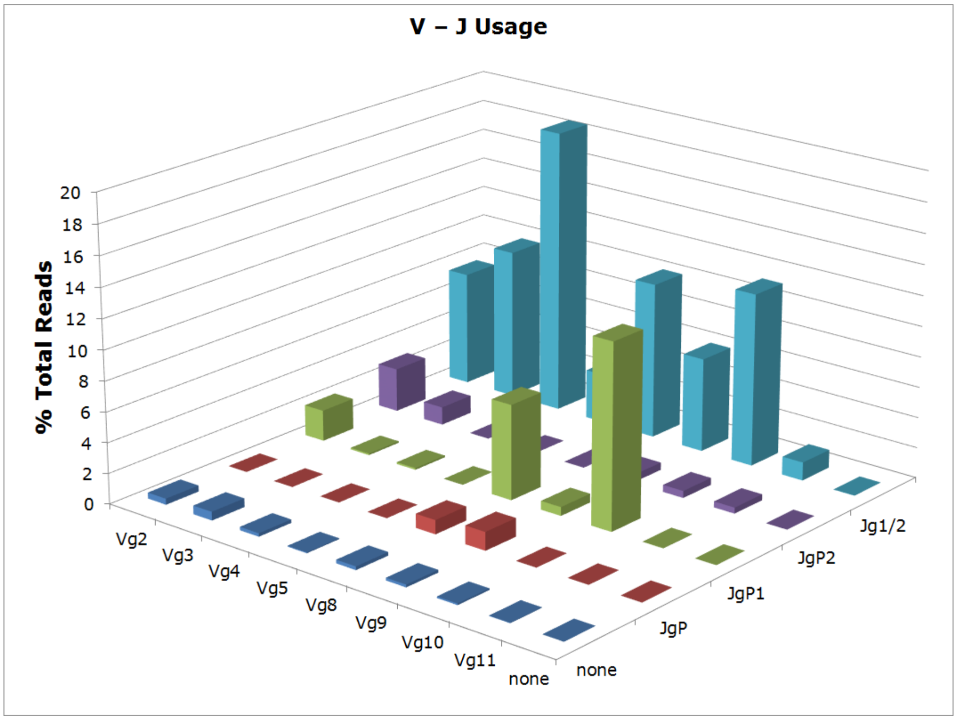
V – J Usage
% Total Reads
20
18
16
14
12
10
8
6
4
2
0
Vg2
Vg3
Vg4
Vg5
Vg8
Vg9
Vg10
Vg11
none
none
JgP
JgP1
JgP2
Jg1/2

B

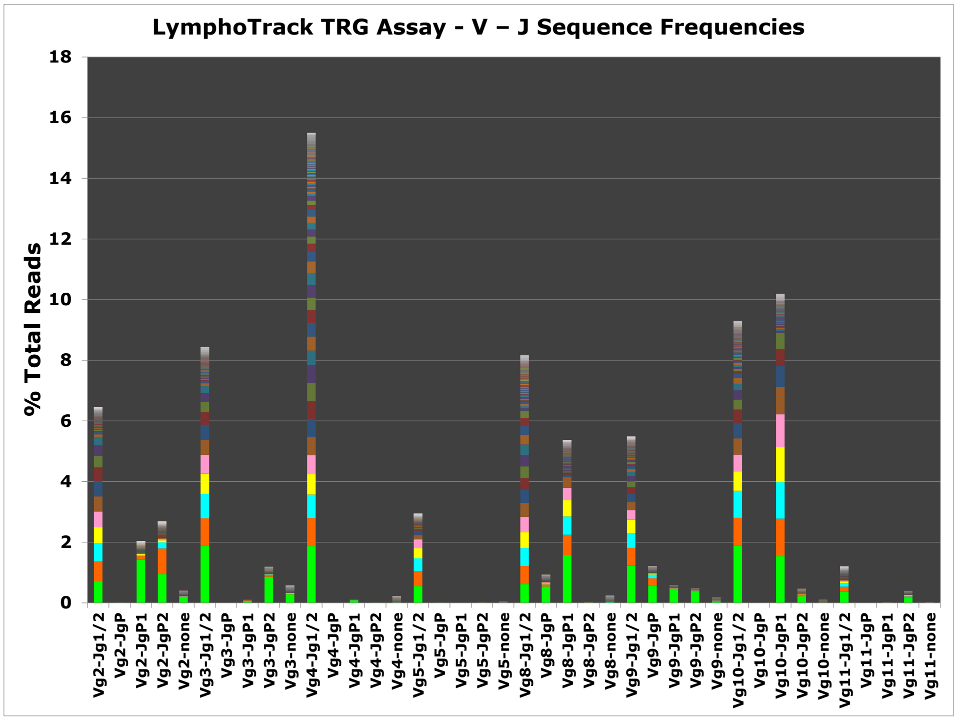
LymphoTrack TRG Assay - V – J Sequence Frequencies
% Total Reads
18
16
14
12
10
8
6
4
2
0
Vg2-Jg1/2
Vg2-JgP
Vg2-JgP1
Vg2-JgP2
Vg2-none
Vg3-Jg1/2
Vg3-JgP
Vg3-JgP1
Vg3-JgP2
Vg3-none
Vg4-Jg1/2
Vg4-JgP
Vg4-JgP1
Vg4-JgP2
Vg4-none
Vg5-Jg1/2
Vg5-JgP
Vg5-JgP1
Vg5-JgP2
Vg5-none
Vg8-Jg1/2
Vg8-JgP
Vg8-JgP1
Vg8-JgP2
Vg8-none
Vg9-Jg1/2
Vg9-JgP
Vg9-JgP1
Vg9-JgP2
Vg9-none
Vg10-Jg1/2
Vg10-JgP
Vg10-JgP1
Vg10-JgP2
Vg10-none
Vg11-Jg1/2
Vg11-JgP
Vg11-JgP1
Vg11-JgP2
Vg11-none

Figure S5. LymphoTrack TRG analysis of (A) V-J usage and (B) V-J sequence frequencies performed on the sample obtained at year 7 from patient #1 showing the same Vg4 Jg1/2 at 18.77% (11,937 reads) and Vg9 Jg1/2 at 6.19% (3,934 reads) as seen in Figure S4, but at a low level, in an otherwise polyclonal background (total read count 63,579).

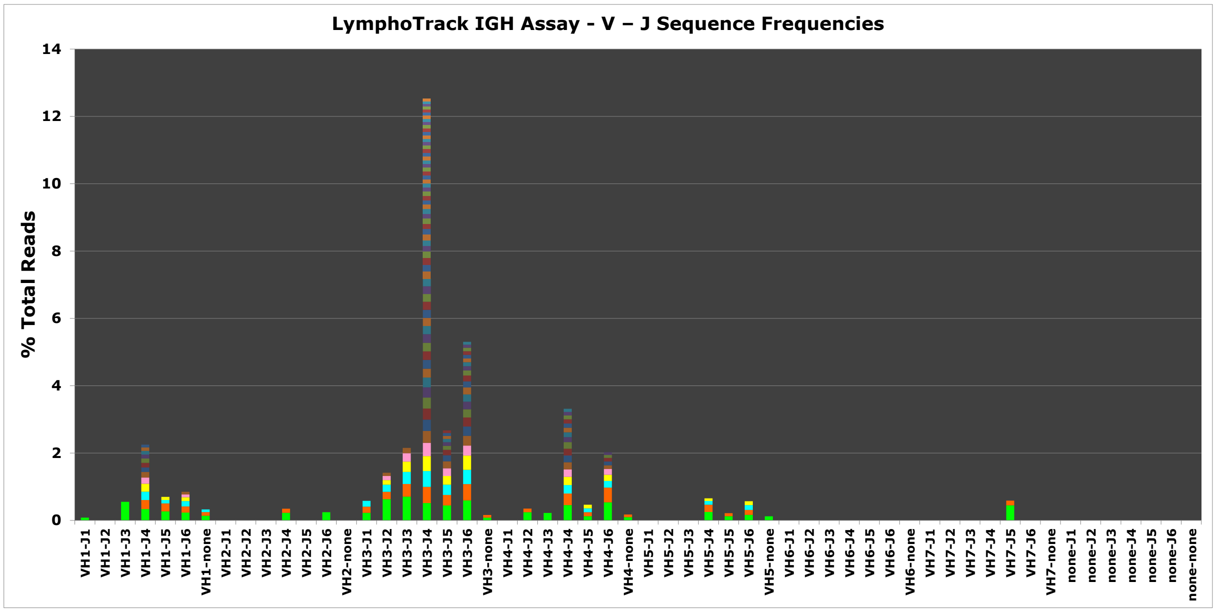

Figure S6. LymphoTrack IGH analysis performed on the sample obtained at year 9 from patient #1 showing a polyclonal IGH (204,929 reads).

For patient #2, the last sample was sequenced with a polyclonal IGH (89,948 reads, Figure S7A) and polyclonal TRG (321,754 reads, Figure S7B) analysis.

A

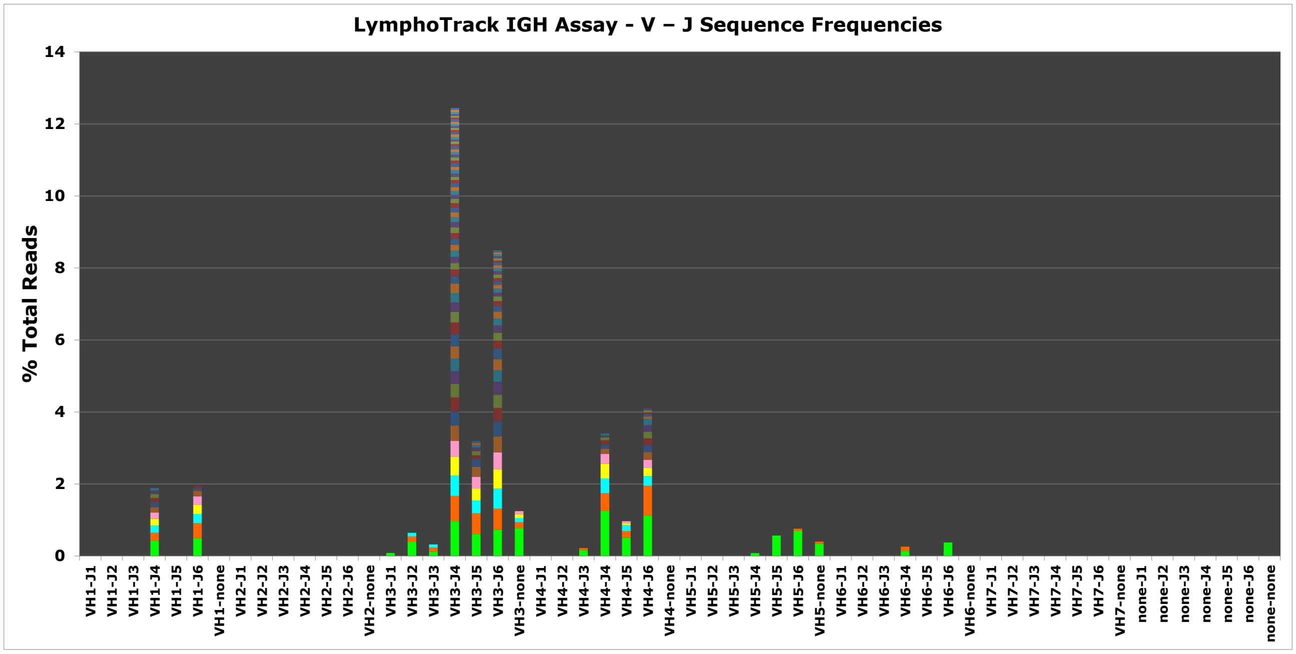

B

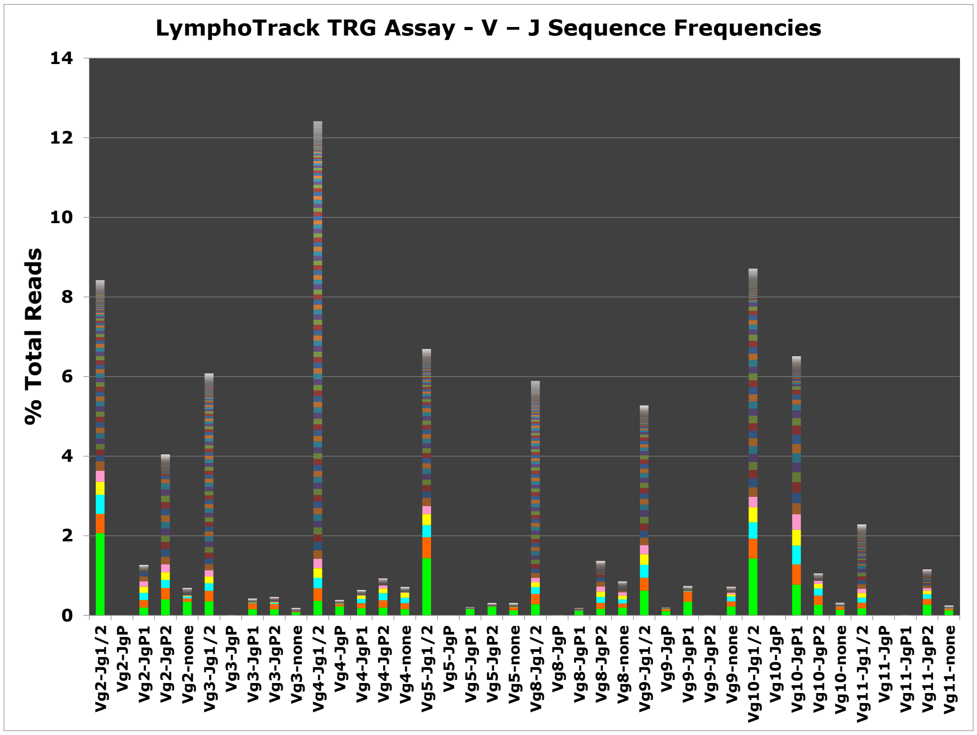

Figure S7. LymphoTrack IGH (A) and TRG (B) analysis performed on the last sample from patient #2 showing a polyclonal IGH (89,948 reads) and a polyclonal TRG (321,754 reads) analysis.
